# Effects of Pulsed Electric Field Technology on Whey Protein Concentrate

**DOI:** 10.3390/molecules31020237

**Published:** 2026-01-10

**Authors:** Elizabeth L. Ryan, Owen M. McDougal

**Affiliations:** 1 Biomolecular Sciences PhD Program, College of Arts and Sciences, Boise State University, Boise, ID 83725, USA; elizabethryan611@u.boisestate.edu; 2 Department of Chemistry and Biochemistry, Food and Dairy Innovation Center, Boise State University, Boise, ID 83725, USA

**Keywords:** PEF, WPC, continuous flow, dairy, spray drying, physical properties, functionality, structural characteristics

## Abstract

Whey protein concentrate (WPC-80) was reconstituted to 10% (*m*/*v*) and pumped through a pulsed electric field (PEF) system using three treatment conditions. The PEF-treated whey solution was assessed for viscosity, whereas dried whey was resolubilized and tested for protein structure integrity by circular dichroism (CD), fluorescence, and differential scanning calorimetry (DSC), and functionality was assessed by measuring solubility, foamability, emulsification, and particle size. PEF treatment resulted in a reduction in apparent viscosity (from 2.74 cP down to 2.57 cP) and particle size (from 325.9 nm down to 297.6 nm), and increased solubility (from 90.41% up to 92.34%) and emulsification stability (from 1727 min up to 4821 min), while emulsification stability decreased initially (from 1.645 m^2^/g to 1.283 m^2^/g) then increased at the high treatment level (1.915 m^2^/g). The foamability and molecular weight profile did not change with PEF treatment. Exposure to PEF resulted in no statistically significant changes to protein structure based on data obtained from CD, fluorescence, or DSC. This study represents the first instance of a WPC-80 being treated with a commercially available, scalable, continuous flow PEF system at a higher concentration (10% *m*/*v*), resulting in favorable changes to the physical and functional properties of the whey solution and dried powder.

## 1. Introduction

Pulsed electric field (PEF) technology has been widely studied across the food industry, but few studies have reported results for PEF technology and whey. Experiments conducted under industrially applicable conditions, with relevant whey concentrations, flow rates, and utilizing commercially available PEF systems, are even less common. There are many experiments that use custom-made PEF systems and widely variable field strength, specific energy, and frequency parameters that demonstrate an effect with dairy solutions, but extrapolation to industrially relevant data to compel technology adoption is far from intuitive.

Whey proteins offer high nutritional value, and their desirable functional attributes, protein quality, bioactive properties, and digestibility make them attractive and versatile ingredients [1,2,3]. Whey proteins are spray-dried to produce a shelf-stable powder for cost-effective storage and transport [4]. PEF technology may improve spray drying efficiency to benefit the dairy industry by increasing plant capacity and reducing energy demand from driers [5].

PEF is a nonthermal technology that applies high voltage electric pulses to samples in a treatment chamber [6]. Both batch and continuous flow PEF systems are available, with continuous flow being most applicable for industrial dairy applications due to the large volume of material being treated [7]. PEF systems that have been used for experiments reported in the literature are primarily custom-made, whereas systems made by Elea Technologies GmbH, Pulsemaster, Heat and Control, and Food Process Solutions (FPS) are commercially available. The PEF parameters, including field strength, specific energy, pulse width, treatment time, frequency, pulse shape, and temperature, have been shown to affect the treatment of food [7]. Depending on the PEF system, these parameters can be changed to optimize treatment of a material to achieve results that range from stress response, electroporation of cells, material softening, facilitating mass transfer, and bacterial inactivation, among others.

Other nonthermal technologies, such as high pressure, cold plasma, irradiation, and ultrasound, have also been explored for their role in processing high-quality and safe foods [8,9]. Compared with other nonthermal technologies, PEF is often considered to be economically and environmentally friendly [10,11]. While there are many benefits to using PEF technology in food processing, a major limitation of the technology is the lack of commercial scale systems and the assumption that results obtained from small-scale systems will translate to commercial-scale applications [12]. Inclusion of PEF in processing has energy requirements, as well as product rheological requirements, that include being a pumpable solution of suitable conductivity.

PEF technology is predominantly used in potato processing, where the electrical pulses electroporate cells, causing the tuber tissue to soften for improved slicing and enhancing the quality of French fries or potato chips by decreasing oil retention during frying, and lowering acrylamide levels in finished products [13,14,15]. The success of PEF to significantly improve potato processing efficiency and product quality has led to the evaluation of the technology throughout food manufacturing for applications associated with plant, animal, and dairy proteins [6,12,16]. The effect of PEF treatment on protein structure appears to be influenced by the type of PEF system, PEF parameters, and the protein being treated, as evidenced by some PEF studies reporting protein structure modification, while others report no structure changes [6,9,12,16].

To date, the application of PEF to bovine milk has been evaluated as an alternative to traditional pasteurization methods [17,18]. While the nonthermal pasteurization of milk is intriguing, current regulatory compliance measures prevent technology adoption for this application. PEF technology has also been explored for its potential to improve sustainability in food processing and possibly even the efficiency to spray dry dairy solutions [10]. PEF treatment of plant, animal, and milk materials has been shown to have a favorable effect on the functional properties of proteins [9,11,17].

A few studies have assessed the effect that PEF treatment of whey solutions has on the physical and functional properties of the proteins [9]. Table 1 provides a compilation of PEF whey studies, where all but one investigation was conducted on whey protein isolate, which is composed of >90% whey protein.

Whereas PEF treatment of non-dairy proteins has been reported, the current investigation is focused on whey solutions. Sui et al. (2011) used a custom-made, flow-cell PEF unit with a four co-field configuration treatment chamber at a diameter of 0.23 cm to PEF-treat a 1% (*m*/*m*) or 10% (*m*/*m*) WPI solution [19]. The PEF parameters used in this study were 35 kV/cm and 131.9 kJ/L, with flow rates of 3.6 L/h for the 1% (*m*/*m*) solution or 14.4 L/h for the 10% (*m*/*m*) solution of WPI. The authors reported no significant effects on physicochemical properties for the WPI. The next study by Xiang et al. (2011) details PEF-treatment of WPI at 3 and 5% (*m*/*v*) with 12–20 kV/cm in a batch PEF system [20]. The WPI showed increased intrinsic and extrinsic fluorescence intensities after PEF treatment, indicating modification to the tertiary protein structure. In a study by Sun et al. (2011), a flow PEF system at 30 mL/min and field strengths of 15 and 30 kV/cm were used to improve the solubility and emulsion properties of WPI-dextran conjugates [21]. Xu et al. (2021) reported the use of a custom-made continuous PEF system to treat a 1% (*m*/*m*) solution of WPI at a field strength of 10 kV/cm to increase succinylation [22]. The succinylated WPI with increased viscosity can be used as an ingredient of ice cream. Most recently, Hu et al. (2024) used a custom-made PEF system, equipped with tandem flow treatment chambers of 1 and 0.4 cm and a flow rate of 1.8 L/h, to treat a 3 mg/mL (0.3% *m*/*v*) solution of whey protein concentrate (WPC-80) [23]. In this study, the field strengths were set within the range of 5–20 kV/cm and specific energies from 0.125 × 10^3^ to 8.04 × 10^3^ kJ/kg. The authors reported decreased viscosity and particle size and increased solubility and emulsifying properties.

The PEF studies with whey that are listed in Table 1 were performed using custom-made, non-commercial systems to expose dilute whey solutions to pulsed electric fields. The narrative shaped from these studies is that PEF treatment of reconstituted whey may alter physical and functional properties, including apparent viscosity, protein solubility, foamability, emulsion stability, and particle size [6,11,19,23]. However, this previous literature lacks many similarities to industrial processing, mainly with custom-made, unscalable PEF systems, slow flow rates, and low reconstituted concentrations. This study aims to move closer to industrial application to justify the move toward industrial adoption.

In the current study, WPC-80 powder was reconstituted to a 10% *m*/*v* solution and pumped through a continuous flow Elea PEF Pilot Dual system equipped with a DN-10 flow cell treatment chamber. The PEF-treated whey solution was assessed for viscosity, whereas dried whey was resolubilized and tested for protein structure integrity by CD, fluorescence, and DSC, and functionality was assessed by measuring solubility, foamability, emulsifying properties, and particle size.

## 2. Results and Discussion

### 2.1. PEF Treatment

Continuous flow PEF treatment of a 10% *m*/*v* WPC-80 solution was performed using three sets of field strength and specific energy PEF parameters that were defined as low (17 kV/cm and 8 kJ/L), medium (19 kV/cm and 12 kJ/L), and high (21 kV/cm and 15 kJ/L), respectively. Previous studies reported positive results on the PEF treatment of milk products with field strengths in the range of 12–26 kV/cm [20,24,25], so we focused on the field strengths within that range. The specific energy has not always been reported in the previous literature or as a consequential parameter determined by other primary parameters. Therefore, the specific energies were chosen based on the maximum frequency (500 Hz) that the Elea PEF Pilot Dual system can achieve with the solution conductivity and product flow rate.

### 2.2. Physical and Functional Properties

Table 2 summarizes the physical and functional property data for whey solution and dried powder following PEF treatment of WPC-80 using relative change, or the proportional change in the PEF-treated sample compared to the control. To monitor the effect of PEF on the whey solution, the pH was taken pre- and post-treatment, with no change from the initial state pH of 5.91. Interestingly, the initial WPC-80 solution conductivity of 2.25 mS/cm was observed to increase by 7.73 to 12.00%, depending on the PEF treatment.

#### 2.2.1. Effect of PEF Treatment on Apparent Viscosity

**Figure 1:**
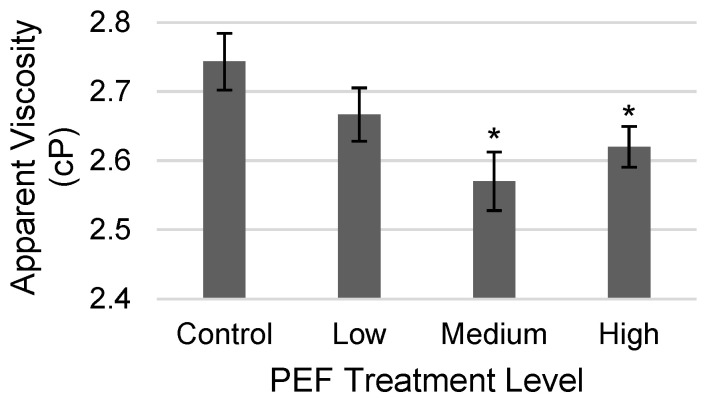
Results of apparent viscosity (cP) for a non-PEF-treated WPC control and PEF-treated WPC samples (Low: 17 kV/cm and 8 kJ/L; Medium: 19 kV/cm and 12 kJ/L; and High: 21 kV/cm and 15 kJ/L). Error bars represent the standard deviations of the measurements in triplicate (*n* = 3). * indicates *p* < 0.05 as compared to the control sample.

The apparent viscosity of PEF-treated WPC solutions at three PEF levels were compared to the non-PEF-treated control (see Figure 1). There was a change in the WPC viscosity for PEF treatment compared to non-PEF control of 2.74 cP, with statistically significant decreases of 6.32% for medium (2.57 cP) and 4.50% (2.62 cP) for high applications measured. The decrease in apparent viscosity obtained as a result of PEF treatment is a very consequential result. Dairy solutions are concentrated through filtration or evaporation prior to spray drying [26]. A limitation to the level of solids in solution that may be obtained is the viscosity of the concentrate that affects the energy required to pump the solution through membranes or into the dryer [27]. An additional benefit of viscosity reduction can be gained when whey powders are resolubilized into beverages, where higher protein content is desired, but thickening is a challenge. Although a lower viscosity was beneficial in this case, certain products, such as ice cream, require increased viscosity that would require different PEF parameters to achieve. In the study by Hu et al. (2024), a similar decrease in the viscosity of whey proteins at 3 mg/mL, when PEF-treated with similar field strengths of 15 and 20 kV/cm, was noted [23]. Despite similar results in another study, the effect of PEF to reduce viscosity in protein-rich dairy solutions has not always been uniformly reported due to a dependence on the intensity of PEF treatment parameters, chamber size, and/or solution flow, and solution composition, including the type of protein and its concentration [11].

#### 2.2.2. Effect of PEF Treatment on Concentrate Solubility

**Figure 2:**
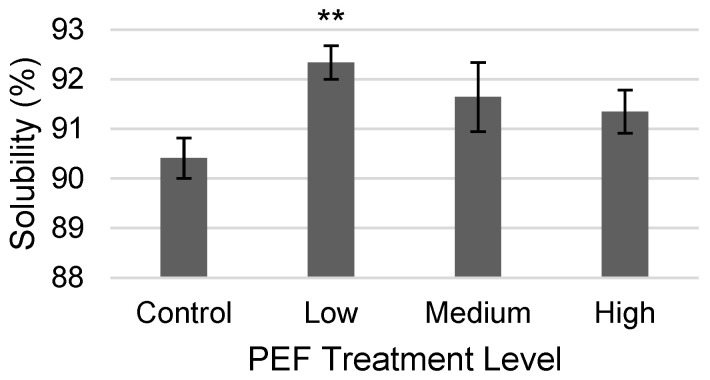
Results of concentrate solubility (%) for a non-PEF-treated WPC control and PEF-treated WPC samples (Low: 17 kV/cm and 8 kJ/L; Medium: 19 kV/cm and 12 kJ/L; and High: 21 kV/cm and 15 kJ/L). Error bars represent the standard deviations of the measurements in triplicate (*n* = 3). ** indicates *p* < 0.01 as compared to the control sample.

Low PEF-treatment conditions increased the solute solubility for the WPC solution by a statistically significant level of 2.1%, compared to the non-PEF-treated, from 90.41% to 92.34% when reconstituted at 15% (*m*/*v*) (Figure 2). The increased solubility is likely because of higher protein–water interactions from the disruption of smaller aggregates, rather than a change in surface charge (see Section 2.2.5). There is precedent for PEF-treated WPI at a concentration of 0.3% (*m*/*v*), flow rate of 30 mL/min, and a field strength of 20 kV/cm to increase the solubility of solids by as much as 10.33% [23]. PEF treatment of 1% (*m*/*v*) WPI at 15 and 30 kV/cm through a flow system (30 mL/min) was also shown to increase solute solubility by facilitating dextran conjugation to whey proteins [21]. It is likely that the modest solubility increase observed in this study is due to the higher flow rate of 6000 mL/min for similar field strengths, resulting in much lower treatment times and less of an effect on the particles in solution.

#### 2.2.3. Effect of PEF Treatment on Foamability

**Figure 3:**
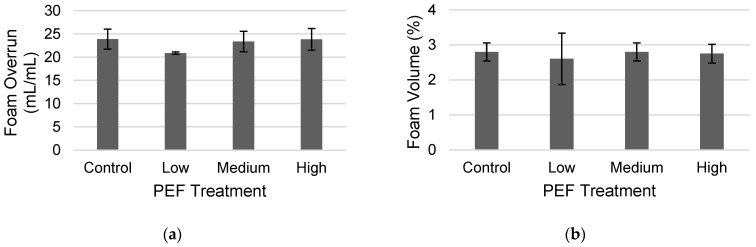
Results of (**a**) foam overrun (FO) and (**b**) foam volume (FV), to represent foam stability over 2 h, for a non-PEF-treated WPC control and PEF-treated WPC samples (Low: 17 kV/cm and 8 kJ/L; Medium: 19 kV/cm and 12 kJ/L; and High: 21 kV/cm and 15 kJ/L). Error bars represent the standard deviations of the measurements in triplicate (*n* = 3).

Whey protein concentrates are used as foaming stabilizers [28]. In the current study, no significant difference in the foam overrun (approximately 24 mL/mL) between the untreated and PEF-treated WPC was observed (Figure 3a), nor was there any change in the foam volume of 2.8%, a measure of stability, over a period of 2 h. (Figure 3b). There was no evidence of insoluble material or aggregates impacting foam properties. Liquid drainage did not occur, indicating that the destabilization mechanism would be through bubble coalescence or bubble disproportionation [29]. The lack of change in foaming properties may be due to the maintenance of the protein-to-fat ratio across treatments or how the proteins interact with each other at the liquid–air interface, which both play a role in foam formation and stability [30,31].

#### 2.2.4. Effect of PEF Treatment on Emulsification

**Figure 4:**
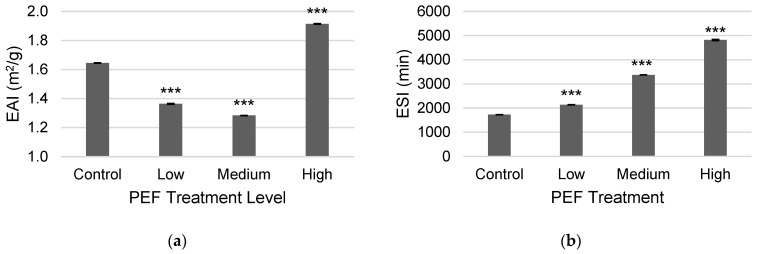
Results of (**a**) emulsifying activity index (EAI) and (**b**) emulsifying stability index (ESI) over 1440 min for a non-PEF-treated WPC control and PEF-treated WPC samples (Low: 17 kV/cm and 8 kJ/L; Medium: 19 kV/cm and 12 kJ/L; and High: 21 kV/cm and 15 kJ/L). Error bars represent the standard deviations of the measurements in triplicate (*n* = 3), and *** indicates *p* < 0.001 as compared to the control sample.

The untreated and PEF-treated WPC were evaluated for the solution’s ability to form an emulsion with oil, known as the emulsifying activity index (EAI) (see Figure 4a). The low and medium PEF treatment levels resulted in a decrease in EAI compared to the untreated control, from 1.645 m^2^/g to 1.364 m^2^/g and 1.283 m^2^/g for the low and medium treatments, respectively, but the high PEF condition exhibited a significant EAI increase to 1.915 m^2^/g. Further assessment of emulsifying stability index (ESI) was conducted, where it was noted that the ESI of PEF-treated WPC significantly increased for each PEF level from low to high, in a stepwise manner, as compared to the control (see Figure 4b). The emulsification stability increased from 1727 min in the control to 2138 min, 3373 min, and 4821 min with low, medium, and high PEF treatments. From these results, PEF may benefit reconstituted whey to emulsify a solution and retain the emulsion over time. Similar results were reported for a 0.3% (*m*/*v*) solution of WPC-80, PEF-treated at 5–20 kV/cm, where low field strength conditions decreased EAI, but increasing field strength increased both EAI and ESI [23].

#### 2.2.5. Effect of PEF Treatment on Particle Size and Zeta Potential

The non-PEF-treated control WPC had a particle size on the order of 325.9 nm, whereas the mean particle size for WPC was significantly lower for all treatment levels, in the range from 297.6 nm to 299.8 nm (see Table 3). The smaller particle size for PEF-treated WPC indicated that not only did protein aggregation not occur, but smaller aggregates that are weakly bound were also disrupted, which is a positive finding for the dairy industry. These smaller aggregate sizes may cause increased protein–solvent interactions, which can positively influence protein viscosity, solubility, and emulsion stability [16]. Particle size reduction for milk proteins by PEF treatment may contribute to improved flowability of powders after processing [32]. The decrease in particle size observed in this study for whey proteins is similar to that reported by Hu et al. (2024), at PEF strengths of 15 and 20 kV/cm [23].

The polydispersity index (PDI), or the uniformity of particle sizes for WPC, showed no significant difference between PEF and the non-PEF-treated control (Table 3).

The zeta potential represents the surface charge of particles in solution, where a larger net magnitude zeta potential is favorable, because it indicates stronger electrostatic repulsion and therefore aggregation is less likely [33]. Similarly to PDI, the zeta potential of WPC was not observed to change due to PEF treatment from approximately −18 mV (Table 3). The inherently large negative zeta potential for untreated and PEF-treated WPC is favorable for emulsion stability [34].

#### 2.2.6. Effect of PEF Treatment on Protein Molecular Weights by Gel Electrophoresis

**Figure 5:**
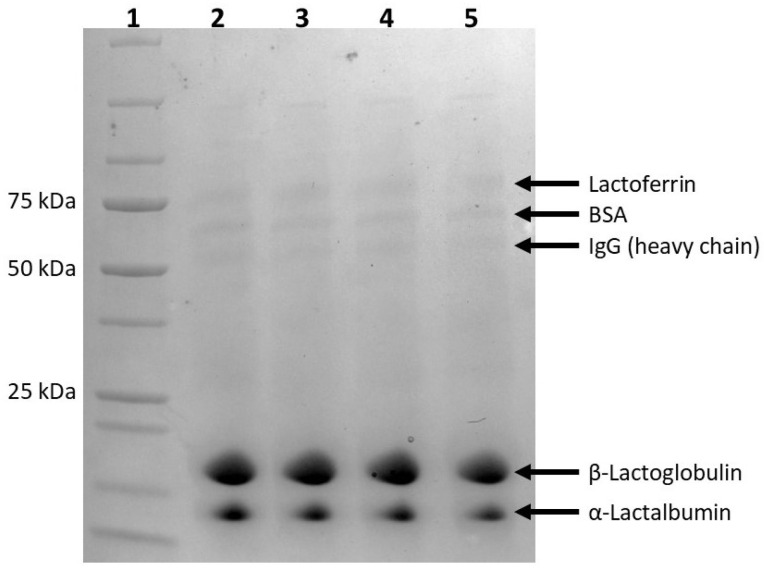
SDS-PAGE depicting protein molecular weights for a non-PEF-treated WPC control and PEF-treated WPC samples (Low: 17 kV/cm and 8 kJ/L; Medium: 19 kV/cm and 12 kJ/L; and High: 21 kV/cm and 15 kJ/L). Lane 1: Protein Standard Ladder; Lane 2: Control; Lane 3: Low; Lane 4: Medium; Lane 5: High.

While protein primary structure is difficult to determine, gel electrophoresis can be used to estimate protein molecular weight. The molecular weight and purity of proteins in the untreated and PEF-treated WPC were visualized through gel electrophoresis (see Figure 5). The major whey proteins observed were β-lactoglobulin and α-lactalbumin with molecular weights of approximately 20 kDa and 14.1 kDa, respectively. Less abundant protein constituents, including the heavy chain of immunoglobulin (IgG), bovine serum albumin (BSA), and lactoferrin were identified due to their molecular weights of ~50 kDa, 66.2 kDa, and 76.5 kDa, respectively. There was no change between the molecular weights and purity of untreated and PEF-treated proteins in WPC. Previous literature has reported similar findings regarding unchanged protein molecular weights with gel electrophoresis after PEF treatment [19,23,35,36,37,38].

### 2.3. Structural Characteristics

#### 2.3.1. Effect of PEF Treatment on Secondary Structure Determined by Circular Dichroism

CD measures the α-helices, β-sheets, turns, and unordered regions that constitute the secondary structure of proteins [8,39]. The CD spectra of untreated and PEF-treated WPC are available in Figure S1. The relative protein secondary structure content, as calculated using Dichroweb, is provided in Table 4. The results show that no statistical significance in the relative content of secondary structures was observed in the current study, using the Elea PEF system.

#### 2.3.2. Effect of PEF Treatment on Tertiary Structure Determined by Intrinsic Fluorescence

The fluorescence spectra of untreated and PEF-treated WPC are shown in Figure S2a. The emission spectra show relatively consistent curves, with the only difference being the intensity of the fluorescence emitted by each treatment. The control has a lower fluorescence intensity across the range of emission wavelengths compared to the PEF-treated samples, where a non-statistically significant increase in fluorescence intensity is observed as PEF treatment levels increase. Figure S2b and Table 5 show the maximum fluorescence intensity associated with each PEF treatment. The fluorescence spectra indicate that protein tertiary structure is likely being retained at every PEF treatment level.

#### 2.3.3. Effect of PEF Treatment on Thermal Stability Determined by Differential Scanning Calorimetry

The melting temperatures of the control and PEF-treated WPC, as determined by DSC, are listed in Table 5. There is no observed change in the melting temperature under any set of conditions, indicating PEF treatment does not impact the heat stability of whey proteins. This observation is also visible in the DSC thermograms, where negative values in the analysis data, in units of µJ/s, indicate a heat-dependent endothermic event (Figure S3). In the study by Sui et al. (2011), WPI was PEF-treated using a continuous flow PEF system at a frequency of 35 kV/cm, and they similarly observed no effect on thermal stability, denaturation temperature, and enthalpy change for the proteins [19].

### 2.4. Correlation Between Protein Structure and Improved Properties

This study investigated whether a modified protein structure was correlated with improvement of physical and functional properties. While previous literature suggests there is a correlation [9,12,16], this study did not show it. Although there were improvements, mainly in the decreased viscosity, increased solubility, increased emulsion stability, and decreased particle size, there were no observed changes in protein structure observed through CD, fluorescence, or DSC. Therefore, this study shows no correlation between modifications in protein structure and improved properties.

## 3. Materials and Methods

### 3.1. Preparation of Whey Protein Concentrate Solution

WPC solutions were prepared by reconstituting ISO Chill^®^ 8000 (Lot number: LN32129390, Agropur, Lake Norden, SD, USA) in water to a concentration of 10% (*m*/*v*). The WPC product had 2.8% ash, 4.5% fat, 9.2% lactose, 6.0% moisture, and 76.2% protein, as determined by the manufacturer. The reconstituted sample (40 L) was homogenized with a paint mixing paddle connected to a drill. The pH of the WPC solution was 5.91, and the conductivity was 2.25 mS/cm. The pH was measured with an advanced automatic potentiometric titrator (Model: HI932, Hanna Instruments, Inc., Woonsocket, RI, USA) and the conductivity was measured with a waterproof pen meter (Model: ST20M-C, OHAUS Corporation, Parsippany, NJ, USA).

### 3.2. Pulsed Electric Field Treatment

The WPC solution was pumped (INOXPA Kiber KSFT progressive cavity pump, Plano, TX, USA) at a flow rate of 6.0 × 10^3^ mL/min (360 L/h) through a continuous flow colinear treatment cell with a 10 mm internal diameter (DN-10) in a PEF Pilot Dual system (Elea Technology GmbH, Quakenbrück, Germany). The sample was treated with field strengths of 17, 19, and 21 kV/cm and specific energies of 8, 12, and 15 kJ/L with a rectangular 6 μs pulse. Field strengths were selected based on positive results from prior research, where milk proteins were PEF-treated [20,24,25]. The specific energies were designated based on the range achievable by the PEF system, given the conductivity of the sample and the flow rate. A non-PEF-treated control sample was collected by running the WPC solution through the PEF system with the generator turned off. Conductivity was measured directly before and after PEF treatment, and viscosity was measured within 24 h.

Samples were freeze-dried with a FreeZone 2.5 Plus benchtop freeze dryer with a Refrigerated CentriVap Vacuum Concentrator (Labconco Corporation, Kansas City, MO, USA) to preserve for further analysis. The moisture content of all four samples was below USDA mandated levels at 5% [40].

### 3.3. Apparent Viscosity

The apparent viscosity was measured by a DVNext cone/plate rheometer (Model: LV-DVNext, AMETEK Brookfield Engineering, Inc., Middleboro, MA, USA) equipped with a cone spindle (Model: CP-40). Liquid WPC solutions (500 μL) were run at 30 rpm for 30 s, and the viscosity in centipoise (cP) and torque in percentage (%) were recorded. For best results, the rpm was determined by finding a speed at which the torque was between 10 and 90%, based on the manufacturer’s recommendation.

### 3.4. Concentrate Solubility

Product solubility was determined using a method adapted from Melchior et al. (2020) [38]. Dried powder was massed and reconstituted to 15% (*m*/*v*), then shaken (Thermo Scientific Solaris Open Air Orbital Shaker, Thermo Fisher Scientific Inc., Waltham, MA, USA) for 1 h at 300 rpm. The sample was centrifuged (Centrifuge 5920R, Rotor FA-6x250, Eppendorf GmbH, Wesseling-Berzdorf, Germany) at 15,000 rcf for 10 min, and the soluble fraction was decanted to retain the pellet. The insoluble fraction was freeze-dried (FreeZone 2.5 Plus, Labconco Corporation, Kansas City, MO, USA) overnight, and the mass of the dried pellet was recorded. Solubility was calculated according to Equation (1):

(1) $$Concentrate\;Solubility\;\left(\%\right)=\;\frac{S-DIF}{S}\times 100\%,$$

where *S* is the initial sample mass and *DIF* is the dried insoluble fraction mass.

### 3.5. Foamability

The foam was prepared by manually shaking 20 mL of a 1 mg/mL WPC solution in a 50 mL closed centrifuge tube for 45 sec at 4 Hz as previously described by Hammershøj et al. (2004) and Schmidt et al. (2018) [31,41]. Foam overrun (FO) was determined by visually measuring the volume of the liquid and foam immediately after the preparation of the foam, and determined by Equation (2):

(2) $$FO\;\left(\frac{mL}{mL}\right)=\;\frac{V_{foam}}{V_{liquid}}$$

The foam was left to sit for 2 h, and the volume of foam and liquid was visually measured at 0, 5, 15, 30, 60, 90, and 120 min. Each sample was run in triplicate. The foam stability was measured with foam volume (FV) and determined by Equation (3):

(3) $$FV\;\left(\%\right)=\;\frac{V_{foam\;(t=120\;min)}}{V_{foam\;(t=0\;min)}}\times 100\%.$$

### 3.6. Emulsification

The emulsifying properties were determined using a method adapted from Khalesi & FitzGerald (2021) [42]. Samples were reconstituted to 0.05 g/mL in nanopure water and stirred until dissolved. The pH was adjusted to 7.0 with 1 M HCl or 1 M NaOH as needed. To ensure dissolution, the sample was heated in a Precision SWB 15 water bath (Thermo Fisher Scientific Inc., Waltham, MA, USA) for 30 min at 50 °C with continuous shaking at 30 rpm. After shaking, the sample temperature was reduced to 4 °C in an ice bath, where it remained for the entirety of the experiment. Sunflower oil with 0.02% (*m*/*v*) sodium azide, an anti-microbial, was used as the oil phase. Commercial sunflower oil was used to replicate food applicability as described in Schmidt et al. (2018) [41]. A 3:1 (*v*/*v*) ratio of protein suspension to sunflower oil was mixed at 16,000 rpm for 1 min using a Fisherbrand 850 Homogenizer (Thermo Fisher Scientific Inc., Waltham, MA, USA). An aliquot from the center layer of the emulsion was taken, stabilized in 0.1% (*m*/*v*) SDS in a 1:40 ratio, and vortexed at 3000 rpm for 30 sec. The emulsifying activity index (EAI) and emulsifying stability index (ESI) were determined by measuring turbidity at 500 nm using a BioTek Epoch 2 Microplate Spectrophotometer (Agilent Technologies, Inc., Santa Clara, CA, USA) with a Type 9 Semi-Micro quartz cuvette (FireflySci, Brooklyn, NY, USA) [41,43,44,45,46]. The EAI and ESI were calculated using Equations (4) and (5), respectively:

(4) $$EAI\;\left(\frac{m^{2}}{g}\right)=\;\frac{2\;\times \;2.303\;\times \;\;A_{0}\;\times \;N}{c\;\times \;\varphi \;\times \;10000}.$$

(5) $$ESI\;\left(min\right)=\frac{A_{0}}{A_{0}-A_{1440}}\times t.$$

where *A*_0_ is the absorbance at 0 min, *A*_1440_ is the absorbance at 1440 min, *N* is the dilution factor (40), *c* is the concentration (0.05), *ɸ* is the oil volume fraction (0.25), and *t* is time (1440 min) [41].

### 3.7. Particle Size and Zeta Potential

Dried powder was reconstituted in water at 0.15% (*m*/*v*) for analysis with dynamic light scattering (DLS) using a Zetasizer Pro (Malvern Panalytical Ltd., Malvern, UK). The DLS instrument has a detection limit of 0.6 nm to 10,000 nm. Samples of 1.0 mL were put in Fisher-brand polystyrene disposable cuvettes (Thermo Fisher Scientific Inc., Waltham, MA, USA) with a 10 mm pathlength. A refractive index of 1.46 was used to measure the total protein. The system equilibrated to 25 °C for 30 sec prior to measurement with the cuvette loaded. All measurements were taken in triplicate. A general-purpose analysis model was used in the ZS XPLORER Software (Version: 4.0.0.683) to determine mean particle size (z-average) and polydispersity index (PDI).

Zeta potential was measured with the same 0.15% (*m*/*v*) samples in a folded capillary zeta cell (Malvern Panalytical Ltd., Malvern, UK). After loading the cuvette, the instrument equilibrated to 25 °C for 120 sec prior to any measurements. A minimum of 10 and a maximum of 50 runs were performed for each sample in triplicate, with at least a 60 s pause between each acquisition. A monomodal analysis model was used to determine zeta potential.

### 3.8. Gel Electrophoresis

Sodium dodecyl sulfate polyacrylamide gel electrophoresis (SDS-PAGE) was performed using 10 mg/mL samples. Reconstituted samples were mixed with 2× Laemmli sample buffer in a 2:1 ratio and denatured by incubating with a reducing agent, β-mercaptoethanol, at a ratio of 1:20 the volume of the sample buffer, at room temperature for 1 h. The samples were then heated to 95 °C for 5 min in a Precision SWB 15 water bath (Thermo Fisher Scientific Inc., Waltham, MA, USA). The samples (10 µL) were loaded into a 4–20% Mini-PROTEAN TGX Precast Protein Gel (Bio-Rad Laboratories Inc., Hercules, CA, USA) alongside 5 µL of Precision Plus Protein All Blue Standards (Bio-Rad Laboratories Inc., Hercules, CA, USA). Electrophoresis was run on a Mini-PROTEAN Tetra Vertical Electrophoresis Cell (Bio-Rad Laboratories Inc., Hercules, CA, USA) at 150 V for 1 h. Gels were stained with Coomassie Brilliant Blue R-250 staining solution and de-stained before they were imaged on a ChemiDoc Go Imaging System (Bio-Rad Laboratories Inc., Hercules, CA, USA).

### 3.9. Circular Dichroism

Circular dichroism (CD) spectra were collected using samples of 0.5 mg/mL on a Jasco J-810 spectropolarimeter (Jasco Corp., Tokyo, Japan). Spectra were recorded using a wavelength range of 180–275 nm with a bandwidth of 1 nm. A quartz cuvette was used with path length of 0.1 cm and spectra for each sample were collected in triplicate. Relative alpha helix, beta sheet, turn, and unordered content were determined with Dichroweb [47,48,49] using the CONTIN algorithm [50] with Reference Set 4 [51].

### 3.10. Fluorescence Spectroscopy

Front face fluorescent spectroscopy was performed by placing 1 mg/mL of sample into a microcell triangular quartz cuvette with a square base and 10 mm path length for measurement in an Agilent Cary Eclipse spectrofluorometer (Agilent Technologies, Inc., Santa Clara, CA, USA). Spectra were collected in triplicate with an excitation wavelength of 290 nm and an emission wavelength range of 305–400 nm to isolate fluorescence from tryptophan residues [20,52,53]. Emission and excitation slit widths were 2.5 nm, the scan rate was set to 60 nm/min with a data interval of 0.5000 nm, and an averaging time of 0.5000 sec. A Savitsky-Golay filter of 15 was used to smooth the data.

### 3.11. Differential Scanning Calorimetry

Differential scanning calorimetry (DSC) spectra were accumulated using a NanoDSC (TA Instruments, Corp., New Castle, DE, USA). A concentration of 5.36 mg/mL was used to obtain thermograms with a temperature range of 20–100 °C and a scan rate of 1 °C/min; DSC scans were acquired in triplicate [54]. The reference cell contained nanopure water, and the baseline was subtracted using a polynomial. NanoAnalyze software (Version 3.12.5) was used to analyze the thermograms and determine melting points with Gaussian modeling.

### 3.12. Statistical Analysis

All experiments were performed in triplicate, and the results are expressed as mean ± standard deviation, with the error bars representing the standard deviation. Statistical differences were determined using a Student’s t-test with a 95% confidence interval. All data with a *p* < 0.05 when compared to the control sample were determined to be statistically significant. Change was represented as relative change according to Equation (6):

(6) $$Relative\;change\;\left(\%\right)=\;\frac{PEF-Control}{Control}\;\times \;100\%.$$

## 4. Conclusions

This study is the first to evaluate continuous flow treatment of a whey solution through a commercially available PEF system. Reconstituted WPC-80 at 10% (*m*/*v*) was pumped through an Elea PEF Pilot Dual flow cell at a rate of 6.0 × 10^3^ mL/min, using three sets of field strength and specific energy conditions designated as low (17 kV/cm and 8 kJ/L), medium (19 kV/cm and 12 kJ/L), and high (21 kV/cm and 15 kJ/L), respectively. Within the range of PEF applications, the whey solution viscosity appeared to modestly decrease (6.32%) at medium PEF levels and conductivity increased for all treatment conditions. With regard to the dried powder, particle size decreased for all PEF applications, and a moderate increase in solubility (2.13%) at low PEF was observed. Based on these results, processing facilities could tailor their PEF treatment to target the improvement they would most like to occur, but all parameter levels used in this study showed benefits to viscosity, solubility, and particle size. PEF did not change foaming properties or negatively impact protein native structure as assessed by CD, fluorescence, and DSC. The present study demonstrates the potential for PEF to be applied to whey concentrates, using a commercially available PEF system that can be scaled for use in industrial settings. Some limitations of this study include the lack of experimentation with regard to the effect of PEF on gelation, storage stability, and sensory properties, as well as different PEF treatments, such as repeated cycles. Future studies should explore industrially relevant application parameters, such as concentrated whey from the processing line prior to spray drying rather than reconstituted powders, and larger treatment flow cells that can facilitate industrially applicable capacity.

## 5. Patents

A patent has been filed on the continuous flow process of PEF applied to dairy solutions on September 16, 2025, and assigned Provisional Patent Application no: 63/882,555 titled, “Continuous Flow Pulsed Electric Field (PEF) Treatment of Dairy Solutions.”

## Figures and Tables

**Table 1:** Studies that report pulsed electric field (PEF) application to whey solutions.

**Whey** **Solution**	**Reconstituted Concentration**	**PEF System**	**PEF Conditions**	Significant Results *	**Citation**
WPI	1% or 10% (*m*/*m*)	Custom, flow-cell with 4 co-field treatment chambers of 0.23 cm at 60 mL/min	35 kV/cm, bipolar square pulse, 2 µs pulse width, 100 Hz frequency, 19.2 µs treatment time, 131.9 kJ/L	No significant changes to physicochemical properties	[19]
WPI	3% or 5% (*m*/*v*)	Custom, batch system with 0.7 cm treatment chamber gap	12–20 kV/cm, 10–30 pulses, 0.5 Hz frequency	FI ↑	[20]
WPI	1% (*m*/*v*)	Custom, flow-cell system with 2 parallel plate treatment chambers at 30 mL/min	15 and 30 kV/cm, square-wave pulse, 25 µs pulse duration, 1.04 kHz frequency, 7.35 ms treatment time	Dextran conjugation ↑ S ↑ EAI ↑ and ESI ↑ Helices and turns ↓ sheets and random coil ↑	[21]
WPI	1% (*m*/*m*)	Custom, continuous system with 0.3 cm electrode distance	10 kV/cm, bipolar square wave, 1 kHz frequency, 40 ms holding time	Succinylation degree ↑ Helices ↓, sheets, turns, and random coil ↑ FI ↓	[22]
WPC-80	3 mg/mL 0.3% (*m*/*v*)	Independently designed with flow-through tandem parallel electrodes (1 and 0.4 cm) at 30 mL/min	5–20 kV/cm, 2–8 ms treatment times, 0.125 × 10^3^ to 8.04 × 10^3^ kJ/kg, 50 Hz frequency, 10 µs pulse width	AV ↑ & S ↑ EAI ↑ & ESI ↑ PS ↓ & ZP ↑ Helices ↓ random coil ↑ FI ↓	[23]

* FI = fluorescence intensity; S = solubility; EAI = emulsifying activity index; ESI = emulsifying stability index; AV = apparent viscosity; PS = particle size; ZP = zeta potential. ↑ = increase; ↓ = decrease.

**Table 2:** The effect of PEF treatment on various physical and functional properties of reconstituted whey protein concentrate (WPC). PEF treatments are labeled as Low (17 kV/cm and 8 kJ/L), Medium (19 kV/cm and 12 kJ/L), and High (21 kV/cm and 15 kJ/L). The change was measured as a relative change and represents the average.

Physical/Functional Property	Relative Change (%) ^†^		
	Low	Medium	High
Conductivity (mS/cm)	↑ 7.73	↑ 10.67	↑ 12.00
Apparent Viscosity (cP)	↓ 2.79	↓ 6.32 *	↓ 4.50
Solubility (%)	↑ 2.13 **	↑ 1.36	↑ 1.04
Foam Overrun (mL/mL)	↓ 12.66	↓ 2.15	↓ 0.26
Foam Volume (%)	↓ 7.02	N/C	↓ 1.74
Emulsifying Activity Index (m^2^/g)	↓ 17.08 ***	↓ 22.01 ***	↑ 16.41 ***
Emulsifying Stability Index (min)	↑ 23.80 ***	↑ 95.43 ***	↑ 179.20 ***
Particle Size (nm)	↓ 8.70 *	↓ 8.38 *	↓ 8.01 *
Zeta Potential (mV)	↑ 2.17	↓ 0.48	↓ 2.45

* indicates *p* < 0.05, ** indicates *p* < 0.01, and *** indicates *p* < 0.001 as compared to the control sample; *n* = 3 for all measurements listed with the exception of conductivity, where n = 1; N/C = no change. ↑ = increase; ↓ = decrease. **^†^** $Relative\;Change\;(\%)=\;\frac{PEF-Control}{Control}\times 100\%$.

**Table 3:** Results of particle size, polydispersity index (PDI), and zeta potential for a non-PEF-treated control and PEF-treated WPC samples (Low: 17 kV/cm and 8 kJ/L; Medium: 19 kV/cm and 12 kJ/L; and High: 21 kV/cm and 15 kJ/L).

PEF Treatment	Particle Size (nm)	PDI	Zeta Potential (mV)
Control	325.9 ± 6.4	0.3616 ± 0.0367	−18.13 ± 0.52
Low	297.6 ± 3.5 *	0.2899 ± 0.0021	−18.52 ± 0.63
Medium	298.6 ± 5.9 *	0.2801 ± 0.0148	−18.04 ± 0.34
High	299.8 ± 2.7 *	0.2648 ± 0.0145	−17.68 ± 0.41

* indicates *p* < 0.05 as compared to the control sample (*n* = 3).

**Table 4:** Results of secondary structure content (%) determined by circular dichroism (CD) for a non-PEF-treated control and PEF-treated WPC samples (Low: 17 kV/cm and 8 kJ/L; Medium: 19 kV/cm and 12 kJ/L; and High: 21 kV/cm and 15 kJ/L). Measurements were taken in triplicate (*n* = 3).

PEF Treatment	Secondary Structure Content (%)			
	α-Helix	β-Sheet	Turns	Unordered
Control	12.5 ± 1.1	31.4 ± 1.2	22.1 ± 1.3	33.9 ± 1.0
Low	11.5 ± 1.4	30.0 ± 2.3	23.5 ± 1.4	35.0 ± 2.3
Medium	11.7 ± 0.4	32.5 ± 1.7	22.6 ± 0.1	33.2 ± 1.5
High	11.0 ± 1.5	28.7 ± 2.3	24.0 ± 1.5	36.2 ± 2.2

**Table 5:** Results of maximum fluorescence intensity (au) determined by fluorescence intensity and melting temperatures (°C) determined by differential scanning calorimetry (DSC) for a non-PEF-treated control and PEF-treated WPC samples (Low: 17 kV/cm and 8 kJ/L; Medium: 19 kV/cm and 12 kJ/L; and High: 21 kV/cm and 15 kJ/L). Measurements were taken in triplicate (*n* = 3).

PEF Treatment	Maximum Fluorescence Intensity (au)	Melting Temperatures (°C)	
		Peak 1	Peak 2
Control	17.62 ± 0.27	69.7 ± 0.3	85.7 ± 0.3
Low	17.63 ± 0.10	69.5 ± 0.1	85.9 ± 0.1
Medium	17.97 ± 0.17	70.0 ± 0.1	85.9 ± 0.1
High	18.25 ± 0.48	68.9 ± 0.3	85.7 ± 0.3

## Data Availability

The raw data supporting the conclusions of this article will be made available by the authors on request.

## Supplementary Materials

The following supporting information can be downloaded at: https://www.mdpi.com/article/10.3390/molecules31020237/s1, Figure S1. Circular dichroism spectra; Figure S2. Fluorescence spectra and maximum fluorescence intensities; Figure S3. Differential scanning calorimetry thermograms.

## Abbreviations

The following abbreviations are used in this manuscript:

PEF	Pulsed electric field
WPC	Whey protein concentrate
*m*/*v*	Mass per volume
kV/cm	Kilovolt per centimeter
kJ/L	Kilojoule per liter
CD	Circular dichroism
DSC	Differential scanning calorimetry
*m*/*m*	Mass per mass
WPI	Whey protein isolate
L/h	Liter per hour
mg/mL	Milligram per milliliter
kJ/kg	Kilojoule per kilogram
mL/min	Milliliter per minute
µs	Microsecond
Hz	Hertz
FI	Fluorescence intensity
ms	Millisecond
S	Solubility
EAI	Emulsifying activity index
ESI	Emulsifying stability index
AV	Apparent viscosity
PS	Particle size
ZP	Zeta potential
mS/cm	MilliSiemens per centimeter
cP	Centipoise
mV	Millivolt
PDI	Polydispersity index
SDS-PAGE	Sodium dodecyl sulfate page electrophoresis
kDa	Kilodalton
IgG	Immunoglobulin G
BSA	Bovine serum albumin
µJ/s	Microjoule per second
au	Arbitrary units
rpm	Revolutions per minute
rcf	Relative centrifugal force
g/L	Gram per liter
FO	Foam overrun
FV	Foam volume
g/mL	Gram per milliliter
*v*/*v*	Volume per volume
DLS	Dynamic light scattering
mm	Millimeter
V	Volt
°C/min	Degrees Celsius per minute
