# Supplementary material for: Effects of Pulsed Electric Field Technology on Whey Protein Concentrate
Source: Molecules. 2026 Jan 10;31(2):237. doi: 10.3390/molecules31020237 (PMC12844111; doi:10.3390/molecules31020237)
Supplement: Supplementary file 1 [file molecules-31-00237-s001.zip › molecules-4049338-supplementary.pdf]

Supplementary Materials: Effects of Pulsed Electric Field Technology on Whey Protein Concentrate

Elizabeth L. Ryan [1] and Owen M. McDougal [2,]

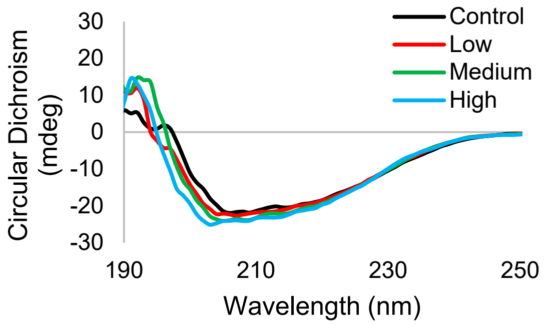

Figure S1. Circular dichroism (CD) spectra for a non-PEF-treated WPC control and PEF-treated WPC samples (Low: 17 kV/cm & 8 kJ/L; Medium: 19 kV/cm & 12 kJ/L; and High: 21 kV/cm & 15 kJ/L).

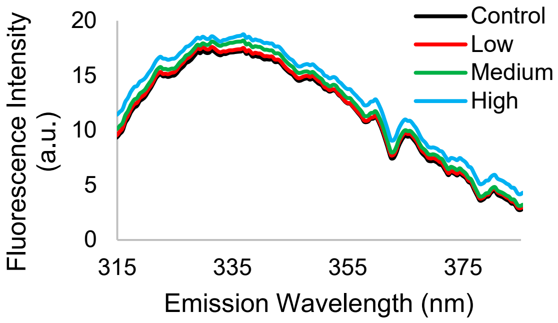

(**a**)

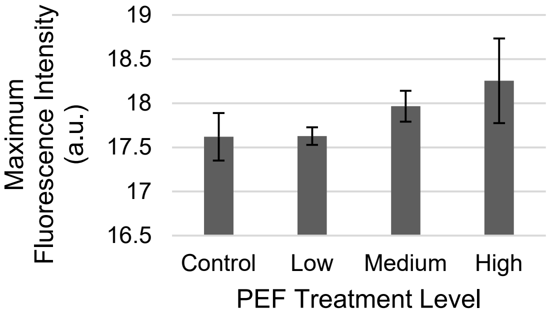

(**b**)

Figure S2. (a) Fluorescence spectra and (b) maximum fluorescence intensities for a non-PEF-treated WPC control and PEF-treated WPC samples (Low: 17 kV/cm & 8 kJ/L; Medium: 19 kV/cm & 12 kJ/L; and High: 21 kV/cm & 15 kJ/L). Error bars represent the standard deviations of the measurements in triplicate.

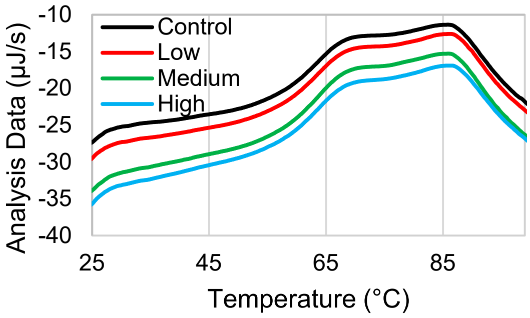

Figure S3. Thermograms for a non-PEF-treated control and PEF-treated WPC samples (Low: 17 kV/cm & 8 kJ/L; Medium: 19 kV/cm & 12 kJ/L; and High: 21 kV/cm & 15 kJ/L) determined by differential scanning calorimetry (DSC). Measurements were done in triplicate and represented as mean ± standard deviation.
